# Inhibition of Glycine Re-Uptake: A Potential Approach for Treating Pain by Augmenting Glycine-Mediated Spinal Neurotransmission and Blunting Central Nociceptive Signaling

**DOI:** 10.3390/biom11060864

**Published:** 2021-06-10

**Authors:** Christopher L. Cioffi

**Affiliations:** Departments of Basic and Clinical Sciences and Pharmaceutical Sciences, Albany College of Pharmacy and Health Sciences, Albany, NY 12208, USA; christopher.cioffi@acphs.edu; Tel.: +1-518-694-7224

**Keywords:** glycine, GABA, glycine transporter (GlyT), glycine receptor (GlyR), nociceptor, *N*-methyl-d-aspartate (NMDA) receptor, spinal cord, dorsal horn, hyperalgesia, allodynia, inflammatory pain, neuropathic pain

## Abstract

Among the myriad of cellular and molecular processes identified as contributing to pathological pain, disinhibition of spinal cord nociceptive signaling to higher cortical centers plays a critical role. Importantly, evidence suggests that impaired glycinergic neurotransmission develops in the dorsal horn of the spinal cord in inflammatory and neuropathic pain models and is a key maladaptive mechanism causing mechanical hyperalgesia and allodynia. Thus, it has been hypothesized that pharmacological agents capable of augmenting glycinergic tone within the dorsal horn may be able to blunt or block aberrant nociceptor signaling to the brain and serve as a novel class of analgesics for various pathological pain states. Indeed, drugs that enhance dysfunctional glycinergic transmission, and in particular inhibitors of the glycine transporters (GlyT1 and GlyT2), are generating widespread interest as a potential class of novel analgesics. The GlyTs are Na^+^/Cl^−^-dependent transporters of the solute carrier 6 (SLC6) family and it has been proposed that the inhibition of them presents a possible mechanism by which to increase spinal extracellular glycine concentrations and enhance GlyR-mediated inhibitory neurotransmission in the dorsal horn. Various inhibitors of both GlyT1 and GlyT2 have demonstrated broad analgesic efficacy in several preclinical models of acute and chronic pain, providing promise for the approach to deliver a first-in-class non-opioid analgesic with a mechanism of action differentiated from current standard of care. This review will highlight the therapeutic potential of GlyT inhibitors as a novel class of analgesics, present recent advances reported for the field, and discuss the key challenges associated with the development of a GlyT inhibitor into a safe and effective agent to treat pain.

## 1. Introduction

The prevalence of chronic pain within the worldwide population ranges from 13% to 25% and the average estimated prevalence of chronic widespread pain (CWP), a more severe form of chronic pain, ranges from 10% to 15% [1]. Approximately 116 million adults in the United States (US) suffer from chronic pain, which presents an incidence rate greater than that for sufferers of cancer, heart disease, and diabetes combined [2]. Estimated associated US annual costs attributed to chronic pain range from a staggering $560 to $635 billion and are projected to escalate [2]. Yet, despite the high level of prevalence and enormous socioeconomic burden incurred, pharmacological treatment of chronic pain remains limited as it is often refractory to currently available analgesics (e.g., NSAIDs, anti-convulsants, antidepressants, topical agents, *N*-methyl-d-aspartate (NMDA) receptor antagonists, and opioids). Furthermore, many of these agents produce modest efficacy for responsive individuals while also inducing severe dose-limiting side effects or presenting a risk of adverse gastrointestinal effects, tolerance, addiction, and abuse. The dearth of effective analgesics has led to an overreliance on opioid medications, which are now the most commonly prescribed class of medications in the US and are fueling a national epidemic of overdose deaths and addictions. Thus, the discovery and development of new analgesics that are more effective and devoid of abuse and addiction liabilities remains a critical unmet need.

Recent insights into the physiological adaptations underlying chronic pain have encouraged efforts to discover new classes of pain-relieving drugs that can selectively target these mechanisms. Among the myriad of cellular and molecular processes identified as contributing to pathological pain, disinhibition of spinal cord nociceptive signaling to higher cortical centers has been discovered to play a critical role. Specifically, impaired glycinergic neurotransmission develops in the dorsal horn of the spinal cord in neuropathic pain models and is an important CNS mechanism causing mechanical hyperalgesia (amplified pain signaling) and allodynia (a painful response to normally innocuous stimuli) [3,4,5,6,7,8,9,10,11,12,13,14]. Thus, it has been hypothesized that agents capable of augmenting glycinergic tone within the dorsal horn may be able to obtund aberrant nociceptor signaling to the brain and serve as a novel class of analgesics. Drugs that enhance dysfunctional and impaired glycinergic transmission in pathological pain states, and in particular inhibitors of the glycine transporter-1 (GlyT1) and glycine transporter-2 (GlyT2), are generating interest as they could offer an innovative approach to induce analgesia via direct modulation of a central gating mechanism controlling nociceptive signaling within the spinal cord [15,16,17,18,19]. Indeed, GlyT1 and GlyT2 inhibitors have demonstrated efficacy in several preclinical models of pain. Such agents could potentially provide an important advance for patients and an opportunity to address the global opioid public health crisis by delivering safe and effective non-opioid analgesics with a mechanism of action that is differentiated from and without the adverse side effects associated with current standard of care.

## 2. Spinal Glycinergic Neurotransmission and Pain Signaling Control

**Figure 1:**
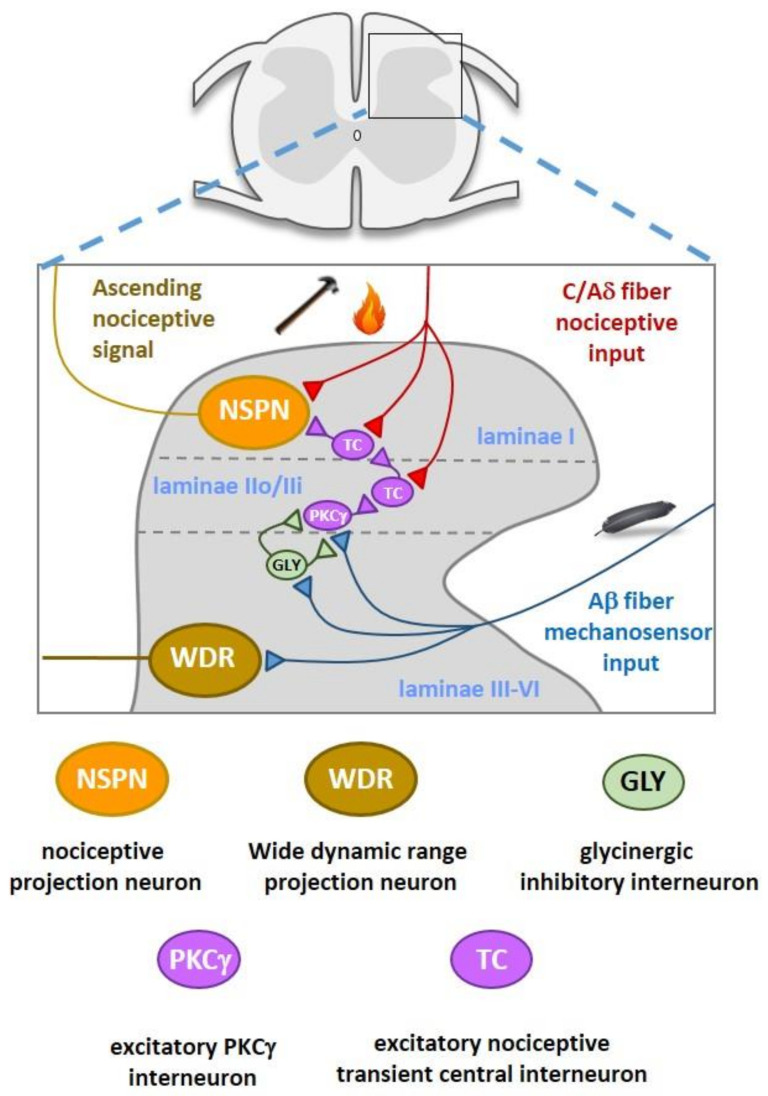
Glycinergic interneurons within the deep dorsal horn (laminae III) are part of a feed-forward inhibitory circuit that prevents innocuous Aβ fiber input from driving nociceptive pathways. Loss of segmental glycinergic inhibitory control via application of strychnine, nerve injury, or targeted ablation and silencing allows low threshold Aβ mechanosensor input to drive NS secondary order projection neurons, resulting in allodynia.

Primary afferent nociceptor and innocuous input is segregated within the dorsal horn and relayed to the higher central nervous system (CNS) via a complex interplay between nociceptive-specific (NS) and wide dynamic range (WDR) projection neurons, excitatory and inhibitory interneurons, and supraspinal pathways (Figure 1). Inhibitory GABAergic and glycinergic interneurons, together with supraspinal descending inhibitory networks, maintain a physiological level of pain sensitivity by controlling or “gating” the intensity and modality of NS projection input to the brain [20,21]. Impaired function of these systems leads to disinhibition of nociceptive drive, which contributes to neuropathic and inflammatory chronic pain. Glycinergic interneurons are highly abundant in the deep dorsal horn of lamina III [10,11,12,13,14]. Glycinergic interneurons within the deep dorsal horn that innervate PKCγ excitatory interneurons are part of a feed-forward inhibitory circuit that prevents innocuous Aβ fiber input from driving nociceptive pathways. Presynaptic and feedforward inhibition of excitatory radial interneurons in inner lamina II (IIi) arises from glycinergic interneurons in lamina III [10,11,12,13,14]. Because both levels receive strong inputs from low threshold Aβ mechanosensors, glycinergic signaling is necessary to prevent innocuous sensory information from invading pain transmitting NS neurons in lamina I. Loss of segmental glycinergic inhibitory control via application of strychnine, nerve injury, or targeted ablation and silencing allows low threshold Aβ mechanosensor input to drive NS secondary order projection neurons, resulting in allodynia. There is compelling evidence that reduced glycinergic signaling in the dorsal horn produces mechanical hyperalgesia and allodynia and develops into chronic neuropathic and inflammatory pain states [3,4,5,6,7,8,10,11,12,13,14].

Using a transgenic mouse line expressing a bacterial artificial chromosome (BAC)-Cre driven by the GlyT2 promoter (GlyT2::cre), Zeilhofer et al. conducted a series of experiments that involved precise ablation, silencing, or activation of glycinergic interneurons in the deep dorsal horn of lamina III [22]. The group reported significantly reduced inhibitory post-synaptic currents (IPSCs) in transverse spinal cord slices from mice with locally ablated or silenced spinal glycinergic interneurons (GlyT2+::cre) relative to negative controls (GlyT2−::cre) using AAV virus-mediated delivery of diphtheria and tetanus toxins, respectively. In addition, ablation or silencing of these interneurons induced long-lasting thermal (heat and cold) and mechanical hyperalgesic pain behaviors and signs of spontaneous discomfort that included flinching, licking/biting, and limb guarding/protection [22]. Conversely, exogenous pharmacogenetic activation of these interneurons with clozapine-*N*-oxide (CNO) mitigated neuropathic hyperalgesia caused by chronic constriction injury (CCI) of the sciatic nerve. The findings from this study provide compelling evidence that glycinergic neurons of the dorsal horn serve as critical elements of a spinal gate for pain and itch signaling to higher cortical centers [22].

Changes in GlyR expression and function have been correlated with altered glycinergic transmission and pain behaviors. GlyR inhibition via administration of sub-convulsive doses of strychnine has been widely reported to induce numerous pain and recurring stereotypic behaviors in rats [23,24,25]. Electrophysiological studies have shown that strychnine induces these behaviors via disinhibition of glycine input on low-threshold Aβ-mediated stimuli that in turn activate NS projection neurons. Furthermore, strychnine was also found to attenuate the analgesic effects of intrathecal (i.t.) administered glycine toward mechanical allodynia in spinal nerve injury (SNI) rats. Imlach and coworkers found that glycinergic transmission at lamina III to radial cell synapses in lamina II is profoundly reduced in adult SNI rats [26]. The cellular and molecular mechanisms involve loss of spontaneous and electrically evoked neurotransmission onto excitatory radial PKCγ interneurons in lamina II, which is mediated by a switch in the subtype of glycine receptors (GlyRs) expressed on these interneurons to GlyRα2 [26]. The GlyRα2 subtype has been reported to be less responsive to synaptically released glycine than GlyRα1 and GlyRα3. This mechanism, coupled with changes in the chloride anion gradient due to reduced potassium-chloride co-transporter (KCC2) expression [27], as reported in superficial lamina I projection neurons, are likely playing a key role in the loss of glycinergic inhibition in the spinal cord that contributes to neuropathic pain. Additionally, Simpson and Huang report that sciatic nerve constriction in rats leads to a bilateral reduction of GlyRs expressed in the dorsal horn [28]. Lastly, prostaglandin type E2 (PGE_2_) and PKA-dependent phosphorylation of the GlyRα3 subtype, which is highly distributed in the superficial dorsal horn where it is expressed on NS secondary order projection neurons, inactivates the receptor and contributes to chronic inflammatory pain [29].

Two published preclinical studies report that pathological pain states can also effect extracellular glycine levels in the CNS, which may present a contributing factor toward observed diminished spinal glycinergic tone and transmission. Lin and colleagues reported that long-lasting hyperglycemia in rats induced by streptozotocin (STZ) injections caused a decrease in the paw withdrawal latency to mechanical stimuli, which was attributed in part to reduced glycinergic inhibitory control of spinal lamina I neurons [30]. Microdialysis experiments revealed persistent hyperglycemia in STZ-induced diabetic neuropathic pain (DNP) rats caused cerebrospinal fluid (CSF) glycine levels to decrease significantly after an initial transient increase, and this observation correlated with observed reductions in the mean frequency of GlyR-mediated miniature inhibitory post-synaptic currents (mIPSCs) of lamina I neurons in spinal cord slices of DNP rats versus wild-type controls. Importantly, i.t. administration of glycine (10 and 100 µg) diminished tactile pain hypersensitivity in DNP rats [30]. A separate study conducted by Miyazato and coworkers demonstrated that glycine concentrations within the lumbosacral cord of chronic spinal cord injury (SCI) female Sprague Dawley rats was approximately 54% lower relative to intact rats [31].

## 3. The GlyTs as Potential Targets to Treat Pain

Preclinical rodent pain behavioral studies involving i.t. glycine administration have shown that spinal application of the amino acid produces anti-allodynic and anti-thermal hyperalgesic effects without inducing adverse neuromotor or respiratory events [32,33,34]. These findings are attributed in part to augmentation of inhibitory glycinergic neurotransmission and they stimulated research efforts focused on studying analgesic effects produced by pharmacologically increasing extracellular CNS glycine concentrations via inhibition of GlyT-mediated uptake. GlyT1 and GlyT2 are high-affinity SLC6 transporters that are largely responsible for maintaining homeostatic synaptic and extrasynaptic glycine concentrations within the CNS. Both transporters present a 12 transmembrane domain (12TM) architecture and share an approximate 50% amino acid sequence homology [35,36,37,38]. GlyT1-mediated glycine transport is bidirectional and likely operating close to equilibrium. It is also electrogenic, requiring a symporter stoichiometry of two Na^+^ cations and one Cl^−^ anion for every molecule of glycine transported [39]. Unlike GlyT1, GlyT2-mediated transport is unidirectional (from extracellular to intracellular space) and requires a symporter stoichiometry of three Na^+^ cations and one Cl^−^ anion for every glycine molecule transported [39]. The differing symporter stoichiometry and unidirectional transport characteristics presented by GlyT2 allows the protein to maintain a high intracellular glycine pool within the presynaptic neuron (concentrations of 10–20 mM) for optimal recycling into presynaptic vesicles and subsequent synaptic re-release [39].

The GlyT1 expression pattern is diffuse as the transporter is found throughout the CNS and retina, with highest levels of distribution located in caudal areas (brain stem, cerebellum, and spinal cord) and lower levels in the forebrain [36,38,40]. The transporter is also expressed outside of the CNS within the dorsal root ganglion [41] and on erythrocytes [42]. Within the CNS, GlyT1 is predominantly localized on glial cells (largely astrocytes), though it also found on post-synaptic glutamatergic neurons, and co-localized with both GlyR and NMDA receptors where it influences their activity by removing glycine from the synaptic cleft and neighboring extrasynaptic regions [43,44,45,46]. Pharmacological inhibition of GlyT1 results in increased extracellular glycine concentrations in these regions, which in turn can lead to enhanced GlyR function and inhibition of spinal nociceptive transmission. Although glycine, along with d-serine, also serves as a co-agonist at NMDA receptors and its binding may promote pro-algesic excitatory glutamatergic neurotransmission, long-term chronic GlyT1 inhibition in a preclinical neuropathic pain model has been shown to induce downregulation of the receptor NR1 subunit within the spinal cord [47].

GlyT2 expression is more circumscribed as the transporter is only expressed within the CNS and is largely confined to caudal areas [48]. Unlike GlyT1, GlyT2 is exclusively co-localized with GlyRs and restricted to presynaptic glycinergic axon terminals, making the transporter an excellent marker for glycinergic neurons. Furthermore, GlyT2 is abundantly expressed in the spinal cord with highest densities within lamina III of the dorsal horn [49]. GlyT2 regulates GlyR function and inhibitory glycinergic neurotransmission via two mechanisms; i) uptake of synaptic glycine into presynaptic terminals to terminate postsynaptic GlyR signaling, and ii) aforementioned repackaging of glycine into presynaptic vesicles via coordination with the vesicular GABA transporter/vesicular inhibitory amino acid transporter (VGAT/VIAAT) for subsequent synaptic re-release [48,50,51]. Thus, spinal nociceptive signaling can also theoretically be suppressed by inhibiting GlyT2, which can lead to an increase glycine concentrations in the synaptic cleft and enhancement of GlyR-mediated inhibitory neurotransmission.

## 4. GlyT1 Inhibitors Studied in Preclinical Pain Models

**Figure 2:**
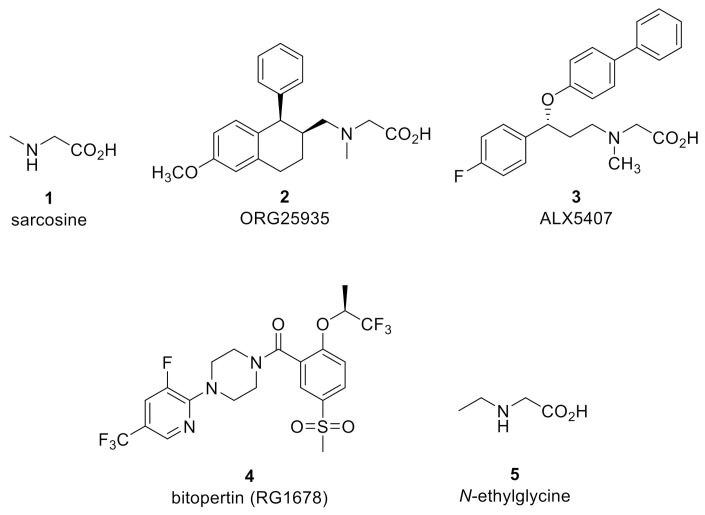
GlyT1 inhibitors studied in preclinical in vivo rodent pain assays.

Five GlyT1 inhibitors, sarcosine (1) (*h*GlyT1 IC_50_ = 91 µM) [29], ORG25935 (2) (*h*GlyT1 IC_50_ ~ 100 nM) [52], ALX5407 (3) (*h*GlyT1 IC_50_ = 3 nM) [53], bitopertin (RG1678) (4) (*h*GlyT1 IC_50_ = 25 nM) [54], and *N*-ethyl glycine (5) [55] (Figure 2), have been studied for analgesic activity in various in vivo rodent pain models. As a class, GlyT1 inhibitors were initially developed for their antipsychotic potential, and sarcosine, ORG25935, and bitopertin were studied clinically for their efficacy to treat persistent negative symptoms or sub-optimally controlled positive symptoms associated with schizophrenia. As previously stated, glycine is an obligatory co-agonist at the NMDA receptor and serves to promote excitatory glutamatergic neurotransmission, which was hypothesized to address glutamatergic hypofunction believed to underlie multiple components of schizophrenia symptomology. Although NMDA receptor activation could potentially be pro-algesic, increased synaptic glycine concentration via GlyT1 inhibition has also been found to prime the NMDA receptor for internalization [56,57]. Indeed, prolonged GlyT1 inhibition has been shown to reduce NMDA receptor expression in the spinal cord [47]. The pharmacological utility of GlyT1 inhibitors has also been explored for several other indications in addition to schizophrenia and pain [58], however, a GlyT1 inhibitor has yet to reach the market for any indication.

The analgesic efficacy of sarcosine and ORG25935 was extensively investigated by Morita and coworkers in a suite of in vivo murine pain assays [59]. Both inhibitors were found to produce significant anti-allodynia effects in the partial sciatic nerve ligation (PSNL) model of neuropathic pain after an initial lag-time of approximately 1.5–2 h post dose (both GlyT1 inhibitors were dosed 0.3 mg/kg, intravenously (i.v.)). The PSNL studies were repeated with NMDA receptor antagonist pre-treatment (L-701,324 or 5,7-dichlorokynurenic acid), which eliminated the lag-time for sarcosine and ORG25935 anti-allodynia effects, suggesting that the GlyT1 inhibitors initially activate excitatory glutamatergic neurotransmission upon increased extracellular glycine concentrations. Activation of these excitatory circuits may be transiently pro-algesic and negating toward any glycinergic inhibitory neurotransmission augmentation [59]. The transience of the observed lag-time could be due in part to NMDA receptor downregulation or desensitization, although further experimental evidence is required to determine the mechanistic pathway(s) involved. Separately, the group also discovered that 1) the analgesic activity of ORG25935 in the PSNL mouse model could be attenuated via strychnine or siRNA knockdown of GlyRα3β, and 2) siRNA GlyT1 knockdown attenuated mechanical allodynia pain behaviors in PSNL mice, providing additional evidence that reducing GlyT1 activity can produce analgesic effects for a chronic pain modality [59]. Morita also reported that spinal application of sarcosine (20 ng, i.t.) or ORG25935 (300 ng, i.t.) reduced mechanical allodynia in mice administered Complete Freund’s adjuvant (CFA) [59]. In addition, GlyT1 inhibitor ORG25935 produced dose-dependent analgesic efficacy in the mouse STZ-DNP model, however, an inverted U-shaped dose–response was observed. This dosing phenomenon is not surprising nor uncommon for the class and has been attributed to transient dizziness and visual disturbances.

Using a mouse femur bone cancer (FBC) model, Motoyama et al. demonstrated that a single i.v. dose of ORG25935 (0.3 mg/kg) produced multi-day improvements in allodynia scores, increased withdrawal thresholds, attenuated guarding behaviors, and reduced limb-use abnormality [60]. This is a significant discovery as bone cancer pain is often refractory toward opioids such as morphine. Similar to the findings reported by Morita, Motoyama also observed that reductions in allodynia scores could be achieved in the mouse FBC model upon siRNA spinal knockdown of GlyT1 [60].

Early sarcosine-derived GlyT1 inhibitor ALX5407 was assessed for analgesic efficacy in the rat chronic constriction injury (CCI) pain model by Hermanns and coworkers [47]. ALX5407 was administered chronically to CCI rats over a period of 14 days via an osmotic infusion pump (0.2, 2, 20, and 200 µg/kg/day, s.c.). Importantly, ALX5407 ameliorated thermal hyperalgesia and mechanical allodynia in a time- and dose-dependent manner and neither respiratory nor neuromotor side effects were observed throughout the duration of the study. Interestingly, a Western blot analysis of the ipsilateral spinal cord revealed significant reductions in NMDA receptor NR1 subunit expression, which may be the result of increased extracellular glycine concentrations leading to increased aforementioned NMDA receptor internalization [56,57] and/or decreased expression levels [47]. These findings provide some support for the hypothesis that the transience of the observed lag-time in the previously described mouse PSNL studies could be due to NMDA receptor downregulation.

A key study reported by researchers at Heinrich-Heine-Universität Düsseldorf highlights the potential therapeutic utility that the GlyT1 inhibitor bitopertin presents for the treatment of chronic neuropathic and inflammatory pain [61]. Bitopertin was initially assessed for efficacy in the mouse CCI model for neuropathic pain whereby a 2.0 mg/kg dose (intraperitoneal, i.p.) induced a significant increase in the reaction threshold to mechanical stimuli 1–6 h post dose. The magnitude and duration of the response was comparable to a 300 mg/kg dose (i.p.) of gabapentin. In addition, the same dose of bitopertin was also found to ameliorate thermal hyperalgesia in the model as a significant increase in the reaction threshold was observed within 2–6 h with the maximal effect observed 6 h after application [62]. Furthermore, the duration of analgesic action was in agreement with the reported rodent PK for the compound (*t*_1/2_ = 4–6 h), providing evidence of a good PK-PD correlation. A lower 0.2 mg/kg dose (i.p.) also produced a significant increase of both the reaction threshold to mechanical stimuli and amelioration of thermal allodynia 2 h post dose in CCI mice. Bitopertin was also assessed for its efficacy at treating chronic inflammatory pain using mice injected with carrageenan [61]. A single 10 mg/kg dose (i.p.) produced significant changes in reaction thresholds to mechanical stimulation and the analgesic effects lasted over a 4 h period. A follow-up dose–response analysis for the model was conducted (0.1, 0.33, 1, 3.3, and 10 mg/kg, i.p.) and a clear dose-dependent anti-allodynic and anti-hyperalgesic effect emerged, demonstrating that bitopertin produces an EC_50_ of 0.6 mg/kg for the anti-allodynic effect and 1.1 mg/kg for the anti-hyperalgesic effect. Importantly, no adverse motor or respiratory effects were observed in a companion open field study (2 mg/kg, i.p.). Concurrently, the team also found that bitopertin significantly and dose-dependently increased CSF glycine levels in mice (10 and 0.6 mg/kg, i.p.) [38]. The CSF study bears significance as it verifies in vivo drug-target engagement with GlyT1 and suggests that CSF glycine may serve as a potential translational biomarker that might provide dose-dependent PK-PD data that can be correlated with analgesic efficacy and may assist with clinical dose predictions. Lastly, the team also explored effects on analgesic activity for neuropathic pain, motor activity, and effects on hemogloblin upon long-term chronic dosing of bitopertin [61]. A continuous supply of bitopertin or vehicle was administered to CCI mice over a time course of 4 weeks (2 mg/kg/day) via implantation of osmotic mini-pumps and sensory testing and open field experiments were performed every 3–7 days. Five days after implantation, a significant increase in the reaction threshold to mechanical stimulation was observed, which persisted as long as the pumps remained implanted and were reversed upon removal of them. Furthermore, no significant differences with hemoglobin levels were observed between the bitopertin and vehicle dosing groups [61]. Lastly, Hermanns and coworkers confirmed that bitopertin also produced significant but transient amelioration of allodynia in CCI rats (1 mg/kg, subcutaneous (s.c.) or oral (p.o.)). The anti-allodynic effects emerged 1 h after administration, but reaction thresholds slowly declined by 2 h and the effect was gone by 24 h post-dose [61]. Collectively, the findings reported by Hermanns provide strong evidence that bitopertin may serve as a novel analgesic for the treatment of chronic neuropathic pain and that clinical investigation of its pain-relieving effectiveness is warranted. Bitopertin has been extensively studied in the clinic, having been evaluated in Phase III trials with schizophrenic patients. The compound has been shown to possess a good safety profile with mild and transient adverse effects (dizziness and visual disturbances). Furthermore, dose-dependent elevations of CSF glycine levels induced by bitopertin have also been observed in humans with the drug, which could provide a potential biomarker to assist with dose predictions and establishing PK–PD relationships that correlate with analgesic activity in a clinical trial for pain [63].

Werdehausen and coworkers reported that *N*-ethyl glycine presents GlyT1 inhibitory activity with no ancillary activity at GlyT2, GlyRs, or NMDA receptors [55]. The compound is a metabolite of lidocaine and may be contributing to the drug’s analgesic effects. Subcutaneous administration of *N*-ethyl glycine to mice injected with CFA induced dose-dependent reductions in inflammatory mechanical hyperalgesia (EC_50_ = 98 mg/kg). A 200 mg/kg dose (s.c.) given to CCI mice also reduced mechanical allodynia and thermal hyperalgesia [55]. In a separate study with carrageenan treated mice, the compound dose-dependently (10, 30, 100, and 1000 µM, s.c.) reduced hyperexcitable dorsal horn WDR secondary order neuron action potentials in response to electrical, thermal, and mechanical stimulation. Furthermore, a 200 mg/kg dose (s.c.) of *N*-ethyl glycine in rats induced a 25% increase in CSF glycine levels versus basal controls, and the timecourse for the observed rat CSF glycine elevation correlated well with the antinociception timecourse observed in the CFA mouse pain model [55], providing additional evidence that CSF glycine levels may serve as a potential translational biomarker for GlyT1 inhibitor pain programs.

## 5. GlyT2 Inhibitors Studied in Preclinical Pain Models

Recent reports suggest that GlyT2 upregulation and enhanced activity occurs in certain pathological pain states. Chronic SCI rats were reported to exhibit significantly higher GlyT2 mRNA levels without changes in GlyT1 or GlyR levels in the L6-S1 spinal cord compared to spinal intact rats [31,62,64]. A separate study conducted by López-Corcuera and colleagues demonstrated how activation of pro-nociceptive P2X_2/3_ receptors with βγ-methylene adenosine 5′-triphosphate induces the up-regulation of GlyT2 transport activity by increasing total and plasma membrane expression and reducing transporter ubiquitination in primary cultures of brainstem and spinal cord neurons [65]. These data suggest that pro-nociceptive action of P2X3 receptors may modulate glycinergic inhibitor neurotransmission and nociceptive singling via enhancing GlyT2 activity, which would theoretically increase glycine uptake and reduce synaptic glycine concentrations. Lastly, Bai et al. recently reported that an upregulation of GlyT2 and a reduction of extracellular glycine levels were observed in the bilateral dorsal horn of the spinal cord at L3-5 of rats in a knee osteoarthritis model, which involved intra-articular injection of monosodium iodoacetate (MIA) into the left knee [66]. The authors reported bilateral ST35 sensitization could be blocked via intraspinal application of GlyT2 short hairpin RNA (GlyT2-shRNA) before MIA injection.

**Figure 3:**
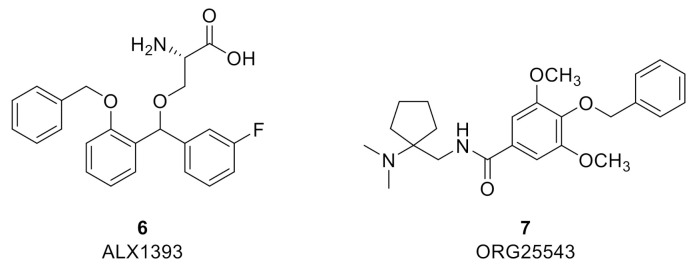
GlyT2 inhibitors ALX1393 and ORG25543.

Due to its restricted distribution, high levels of expression in the deep dorsal horn, selective co-localization with GlyRs, and apparent upregulation in certain pain states, GlyT2 provides a highly attractive target to augment spinal glycinergic neurotransmission and treat chronic pain while potentially avoiding side effects associated with centrally acting analgesics and drugs that target GlyT1 [67]. Multiple high affinity and selective GlyT2 inhibitor chemotypes have been reported to demonstrate efficacy in several preclinical rodent models of various pain modalities. The two most extensively investigated GlyT2 inhibitors to date are ALX1393 (6) (*h*GlyT2 IC_50_ = 100 nM; inhibition of [^3^H]-glycine uptake in stably transfected HEK-293 cells expressing *h*GlyT-2) [45] and ORG25543 (7) (*h*GlyT2 IC_50_ = 16 nM; inhibition of [^3^H]-glycine uptake in CHO cells stably expressing *h*GlyT-2) [68,69] (Figure 3). Both compounds display limitations that preclude their development. However, despite their limitations, they have served as effective and widely used pre-clinical pharmacological tools to help elucidate differences in effects on synaptic glycinergic neurotransmission ex vivo and to determine the potential therapeutic utility of GlyT2 inhibition for diverse pathological pain states in vivo.

ALX1393 is a lipophilic amino acid that also exhibits inhibitory activity at GlyT1 (*h*GlyT1 IC_50_ = 4 µM) and is a poorly CNS permeable (*K*_p, uu_ = 0.036, mouse), likely due to the carboxylic acid functional group it bears. ORG25543 exhibits better CNS permeability than ALX1393 (*K*_p, uu_ = 0.5, mouse) and it does not display ancillary activity at GlyT1. Separate ex vivo electrophysiology studies with the two inhibitors have established that blockade of GlyT2 produces a tonic glycinergic current and enhances glycinergic eIPSCs in lamina II neurons from normal animals. In studies with mouse spinal cord slices, ALX1393 was found to prolong the decay phase of GlyR-mediated evoked inhibitory post-synaptic currents (eIPSCs) without affecting their amplitude. In addition, ALX1393 induced a tonic inward current in the presence of tetrodotoxin. The induced inward tonic currents were reversed with co-application of strychnine. Lastly, although the GlyT1 inhibitor ALX5407 had no statistically significant influences on miniature inhibitory post synaptic currents (mIPSCs), ALX-1393 significantly increased their frequency [70]. Similarly, ORG25543 was found to induce slowly developing GlyR-mediated inward currents and dose-dependent increases in decay time constants of mIPSCs, eIPSCs, and spontaneous inhibitory post synaptic currents (sIPSCs) in ex vivo studies using lamina X neurons from rat spinal cord slices [71]. Furthermore, a significant increase in extracellular glycine levels within the rat spinal cord (337 ± 76% increase relative to basal levels) was observed upon microdialysis perfusion of ORG25543 (10 µM) [71].

The analgesic efficacy of ALX1393 for treating acute (nociceptive) and inflammatory pain was assessed in rats using the hot plate and tail flick tests, a paw pressure test, and the formalin test by Haranishi and coworkers [72]. Intrathecal administration of ALX-1393 prolonged latencies in the hot plate and tail flick assays in a dose-dependent manner and increased vocalization thresholds in the paw pressure test (single dose), and these observed antinociceptive effects could be abolished by co-administration of strychnine. Interestingly, ALX1393 was only found to significantly suppress Phase II pain behaviors in the rat formalin test. The assay produces a biphasic pain response to formalin injection in the hind paw, with Phase I acute nociceptive pain behaviors occurring approximately 10 min post formalin injection and Phase II behaviors beginning approximately 60 min post-injection in response to central sensitization [72]. Phase I analgesia was only observed in response to a 40 µg dose, whereas Phase II dose-dependent analgesic was observed for the 20 and 40 µg doses. These observations for the formalin model may indicate that GlyT2 inhibition is more effective for treating a pain state whereby alterations in spinal glycinergic neurotransmission have had time to occur. Lastly, no adverse motor effects were observed for ALX1393 in the rotarod test at analgesic doses (20 and 40 µg, i.t.). However, a higher 60 µg i.t. dose did induce severe respiratory depression and motor impairment [47], effects observed for the *GlyT1^−/−^* knockout mouse phenotype and for some slowly dissociating GlyT1 inhibitors (e.g., ALX5407) [44]. The fact that ALX1393 is inducing these adverse effects at a high dose could be attributed in part to extensive and prolonged GlyT1 inhibition.

Morita and coworkers also examined the analgesic efficacy of ALX1393 and ORG25543 in the previously described battery of mouse neuropathic and chronic inflammatory pain models [59]. The group reported that i.t. and i.v. administration of either compound significantly and dose-dependently reduced paw withdrawal thresholds (von Frey) for *PSNL* and STZ-induced DNP mice. Furthermore, single-dose spinal application of either compound also produced significant anti-allodynia effects in the mouse CFA model [59]. No adverse effects on locomotor activity, motor behavior, or the righting reflex were observed in any of these studies. Importantly, the analgesic effects observed for ALX1393 and ORG2554 in these assays could be antagonized by co-application of strychnine or by siRNA GlyRα3β knockdown, providing additional evidence that the mechanism of action by which GlyT2 inhibition induces analgesia is via augmentation of spinal inhibitory glycinergic neurotransmission [59].

A separate neuropathic pain study conducted by Hermanns et al. investigated analgesic effects of an acute dose of ALX1393 (10, 50, or 100 µg, i.t.) in the rat CCI model [73]. Interestingly, only the highest dose administered attenuated pain behaviors, however, severe respiratory depression were also observed. These side effects are similar to what was reported by Haranishi and are again attributed to ancillary GlyT1 inhibition. A separate 14-day chronic dosing study with ALX1393 (0.2, 2, 20, and 200 µg/kg/day; s.c. via osmotic infusion pump) and CCI rats showed that the inhibitor produced dose- and time-dependent reductions in thermal hyperalgesia and mechanical allodynia without adverse respiratory or motor effects [47]. Western blot analysis of the ipsilateral spinal cord revealed no changes in GlyT2 expression levels.

In addition to in vivo assays of acute, inflammatory, and surgically-induced neuropathic pain, ALX1393 and ORG2554 have also been reported to produce dose-dependent analgesia in models of herpetic, visceral, and cancer-induced pain. Spinal application of the GlyT2 inhibitor ALX1393, but not the GlyT1 inhibitor sarcosine, dose-dependently ameliorated dynamic and static allodynia in a mouse herpetic and postherpetic pain model involving percutaneous inoculation with herpes simplex virus type-1 (HSV) [74]. Spinal application of ALX1393 also significantly increased the intercontraction interval and the micturition pressure threshold during cystometry and strongly suppressed the micturition reflex in cyclophosphamide (CYP)-treated rats, a model of bladder pain and interstitial cystitis [31,62,64]. Both ALX1393 and ORG2554 have been reported to exhibit dose-dependent and multi-day improvements in allodynia scores in a murine femur bone cancer (FBC) pain model, which are key findings as bone cancer pain is often refractory to opioid treatment. Motoyama and co-workers showed that administration either ALX1393 (0.01 mg/kg, i.v.) or ORG2554 (0.03, 0.1, and 0.3 mg/kg, i.v.) 11 days after NCTC 2472 tumor cell implantation ameliorated tactile allodynia, withdrawal threshold, guarding behavior, and limb-use abnormality [60]. Furthermore, the observed analgesic effects lasted 5–10 days post-dose. Oral administration of ALX1393 (0.3 and 1 mg/kg) was similarly effective. Importantly, experiments involving siRNA knockdown of spinal GlyT2 in FBC mice recapitulated the analgesic effects observed with the GlyT2 inhibitors. Notably, ORG2554 (0.03, 0.1, and 0.3 mg/kg, i.v. or 0.3 and 1.0 mg/kg p.o) also exhibited synergistic effects with sub-therapeutic doses of morphine (0.3 mg/kg, s.c.) and significantly ameliorated pain-like behaviors [60]. These findings are significant as they provide evidence that GlyT2 inhibitors may potentially also potentially provide utility as either stand-alone analgesics or as opioid-sparing agents.

## 6. Mechanism-Based Safety Concerns for GlyT2 Inhibitors

Despite the compelling and promising preclinical proof-of-concept data for ALX1393 and ORG255, perceived on-target liabilities associated with a lethal knockout mouse phenotype have significantly stalled advancement for the field. Neither ALX1391 nor ORG25543 is orally bioavailable and ALX1393 is poorly CNS permeable with only 10-fold selectivity for GlyT2 over GlyT1. ORG25543 causes loss of motor control at high doses, which has been attributed to its slow dissociation and long residence time with subsequent reductions in presynaptic glycine reloading. The adverse effects associated with ORG25543 at high doses mimic the homozygous *GlyT2^−/−^* knockout mouse phenotype. Homozygous *GlyT2^−/−^* knockout mice live to the second postnatal week and present tremor, spasticity, and impaired motor coordination due to insufficient presynaptic reloading of glycine and reduced GlyR neurotransmission [75,76]. Loss-of-function homozygous mice derived from a spontaneous GlyT2 mutations also express an identical hypoglycinergic phenotype [77]. More recently, Marsala and colleagues reported that administration of a GlyT2 antisense oligonucleotide (GlyT2-ASO) in a rat model of spinal transection-induced muscle spasticity and profound spinal hyper-reflexia, which was attributed to a >90% decrease in GlyT2 mRNA and protein within the lumbar spinal cord (as per immunofluorescence staining and Q-PCR analysis) [78]. These in vivo observations correlate with reported ex vivo studies with cultured neurons from rat spinal cord slices, which show how prolonged exposure to very high concentrations of ALX1393 or ORG2554 generates an initial increase in glycinergic neurotransmission followed by significant reduction, presumably resulting from unfavorable disruption of glycine re-supply into presynaptic vesicles. In humans, homozygous or compound heterozygous recessive inheritance carriers of various missense, nonsense, and frameshift GlyT2 (*SLC6A5*) mutations display hyperekplexia, a paroxysmal neurological disorder caused by impaired glycinergic neurotransmission [79,80,81,82]. Notably, hyperekplexia patients also demonstrate increased pain sensitivity and impaired central pain modulation compared to healthy controls, underscoring the important role that glycinergic neurotransmission plays in central pain modulation. Collectively, these preclinical and clinical observations led to the belief that GlyT2 may be an intractable target as inhibition of it could ultimately lead to adverse mechanism-based hyperekplexic effects.

**Figure 4:**
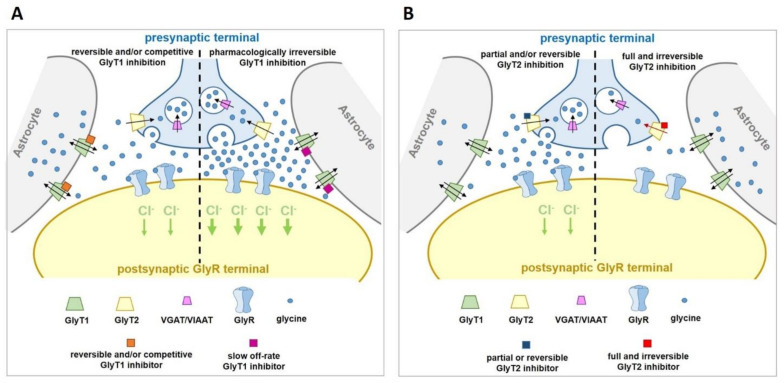
Potential functional consequences of GlyT inhibition. (**A**) Reversible GlyT1 inhibition with rapid dissociation (shown on the left) versus prolonged inhibition via slow dissociation (shown on the right) at an inhibitory glycinergic synapse. A slowly dissociating GlyT1 inhibitor (magenta square e.g., ALX5407) can potentially generate a sustained and excessive increase in synaptic glycine that can ultimately lead to robust and prolonged GlyR activity. This hyperglycinergic state is believed to underlie the severe motor impairment and respiratory depression observed for tight binding GlyT1 inhibitors. Reversible GlyT1 inhibitors (orange square, e.g., bitopertin), that slowed the clearance of synaptic glycine while also allowing for glycine reuptake, were found to exhibit significantly improved safety profiles. (**B**) Partial or reversible GlyT2 inhibition with rapid dissociation (shown on the left) versus full GlyT2 inhibition and slow dissociation (shown on the right) at an inhibitory glycinergic synapse. A slowly dissociating GlyT2 inhibitor (red square e.g., ORG25543) will initially generate an increase in synaptic glycine concentrations but will ultimately prevent glycine reloading of synaptic vesicles and reduce both glycine release and GlyR activity. A partial or reversible inhibitor that rapidly dissociates (blue square) will slow the clearance of synaptic glycine, increase GlyR activity, and also allow for glycine reuptake and reloading of vesicles.

However, there is compelling evidence that partial and/or reversible inhibition of GlyT2 can be both safe and effective for treating pain. Heterozygous *GlyT2^+/^^−^* knockout and mutant mice display no gross anatomical abnormalities, are viable, and are devoid of any of the adverse motor behaviors expressed in the knockout phenotype [75,76,77]. In addition, human heterozygous carriers of *SLC6A5* mutations do not express hyperekplexia [79,80,81,82]. These observations demonstrate that significant but not complete loss of GlyT2 function does not disrupt glycinergic neurotransmission and is well tolerated in rodents and humans. Morita reported that GlyT2 siRNA knockdown in PSNL mice (75% knockdown relative to WT controls) significantly reduced allodynia over a timecourse that correlated with reductions in GlyT2 immunoreactivity, providing corroborating evidence that reduced GlyT2 activity can effectively alleviate pain [59]. Importantly, the siRNA GlyT2 knockdown mice showed no adverse motor effects or any other behaviors observed for GlyT2 KO mice. This study demonstrates that partial GlyT2 reduction appears to be sufficient to slow the clearance of glycine from synapses and enhance glycinergic tone while still allowing sufficient re-uptake to maintain neurotransmission (Figure 4). The overarching question to be addressed for the field is how to pharmacologically reproduce the effects of partial siRNA knock down of GlyT2 and avoid the harmful effects of complete loss of GlyT2 activity as a way of developing novel analgesics without side effects. Thus, approaches to develop partial and/or reversible GlyT2 inhibitors are now emerging.

## 7. Allosteric Partial and Reversible GlyT-2 Inhibitors: Bioactive Lipids

**Figure 5:**
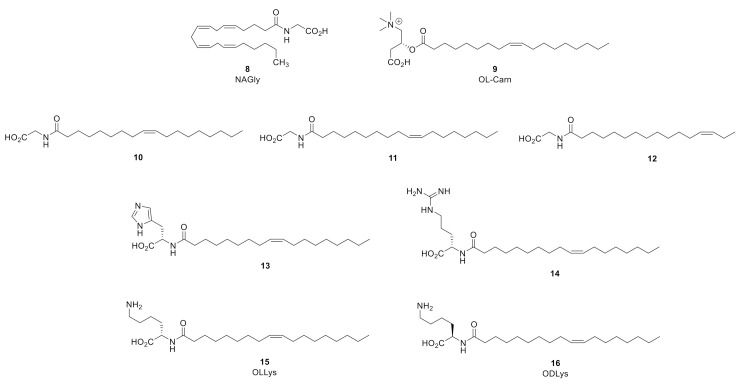
Bioactive lipid GlyT2 inhibitors.

*N*-arachidonyl-glycine (NAGly, 8) (Figure 5) is an endogenous bioactive lipid that is structurally related to the endocannabinoid anandamide and is produced in highest concentrations in the spinal cord. Jeong and coworkers have demonstrated that NAGly is a relatively potent, selective, non-competitive, reversible, and partial inhibitor of GlyT2 inhibitor (GlyT2 IC_50_ = 9.1 ± 3.1 µM) [83,84]. Maximal concentrations of NAGly (30 µM) present partial 87.9 ± 12.5% inhibition in an electrophysiological assay with *h*GlyT2 expressed in *Xenopus laevis* oocytes or HEK293 cells. In general, the electrogenic process of GlyT2 transport allows two-electrode voltage-clamp electrophysiology to be used to measure glycine transport. IC_50_ values and %maximal inhibition for NAGly were assayed by measuring glycine dependent (EC_50_) currents measured at −60 mV in the presence of increasing compound concentrations. Inhibitor concentration responses were measured by cumulative application. The EC_50_ concentration of glycine was applied until a stable level of transport was reached. Increasing concentrations of NAGly were then co-applied with glycine, with each concentration producing a distinct plateau in response. Currents at each of these plateaus were measured and compared to glycine currents in the absence of any inhibitor. The reversibility of NAGly and related bioactive lipid GlyT2 inhibitors was assessed by transport recovery after a washout timecourse using the aforementioned two-electrode voltage-clamp assay. The EC50 concentration of glycine was applied until a stable level of transport was reached, which is denoted as the maximal inward current (I_max_) as this is the inward current recorded in the absence of inhibitor. A GlyT2 inhibitor, such as NAGly, was then co-applied at its IC_50_ concentration with glycine. Once stable inhibition was reached, an assay buffer was then perfused into the recording bath to wash the oocytes. EC_50_ glycine was then reapplied at 5 min intervals over a 30 min (or longer) timecourse to determine the rate of transport recovery, which is indicative of the reversibility of an inhibitor. The transport recovery over the 5 min intervals is represented as the observed inward transport current (I) over the original maximal inward current obtained in the absence of inhibitor (I/I_max_).

Upon additional screening, NAGly exhibited no activity at GlyT1 or the GABA transporter GAT1 [83,84]. However, the bioactive lipid was found to exhibit ancillary GlyR PAM activity. The group also showed that NAGly inhibits glycine transport in the dorsal horn and indirectly stimulates GlyR activity without effecting NMDAR-mediated EPSCs, supporting the hypothesis that selective inhibition of GlyT2 can increase GlyR-mediated inhibitory neurotransmission without glycine spillover into excitatory NMDAR synapses. Furthermore, i.t. or s.c. application of NAGly provides analgesia in rat models of neuropathic and inflammatory pain (formalin test, partial nerve ligation (PNL) and CFA models) without motor side effects, providing in vivo proof-of-concept that partial GlyT2 inhibition is capable of decoupling analgesia from mechanism-based toxicity [84]. Thus, NAGly may form part of an endogenous system for regulating glycinergic neurotransmission in the spinal cord to reduce the perception of pain. These observations opened new avenues of research to identify novel bioactive lipids that partially inhibit GlyT2 to treat pain without adverse motor effects. The Vandenberg group subsequently reported that oleoyl-l-carnitine (OLCarn, 9) is also a selective, non-competitive, and partial GlyT2 inhibitor that was approximately 15-fold more potent than NAGly (*h*GlyT2 IC_50_ = 340 nM) with a maximal inhibition of 60% at 1 µM [84]. Interestingly, OLCarn exhibited a slower onset of inhibition and slower transport recovery in the washout assay relative to NAGly. These findings stimulated further investigation into the structure–activity relationships (SAR) and the putative allosteric GlyT2 binding site for the class.

A novel series of monounsaturated C18 and C16 acyl-glycine lipid analogues were initially synthesized and tested for activity at GlyT2 [85]. All of the compounds were found to be inactive at GlyT1 and an interesting GlyT2 SAR profile surrounding the lipid tail double bond emerged. Of the series, the C18ω8 (10, *h*GlyT2 IC_50_ = 320 nM), C18ω9 (11, *h*GlyT2 IC_50_ = 500 nM), and C16ω3 (12, *h*GlyT2 IC_50_ = 810 nM) were the most potent with maximal inhibition values ranging between 61.3–66.8% in the electrophysiological oocyte assay. The specificity of inhibition of glycine transport for key compounds was also measured using [^3^H]-glycine uptake by oocytes expressing GlyT2 and GlyT1, confirming observations with electrophysiological measurements. A loss of activity at GlyT2 activity was observed for 18-carbon chain congeners of 10 presenting a repositioning of the double bond by more than two bonds from the ω9 position [85]. Shifting the double bond by only one carbon had a less significant effect on GlyT2 potency, but shifting the position of the double bond by one further carbon to either end of the lipid chain abolished inhibitory activity [85]. To further probe the importance of the double bond, the geometric trans-isomers of the cis-C18ω7 and C18ω9-analogues were screened and found to be inactive. These SAR trends suggest there is a selective hydrophobic binding pocket on GlyT2 which can only accommodate the cis-conformations of the lipid tail moiety and that the placement of the double bond within the lipid chain was limited to a precise centralized region within the pocket. Follow-up SAR with NAGly bioactive lipids presenting a 16-carbon lipid and a double bond at any of the ω3, ω5, ω6, ω7, ω9, ω11, or ω12 positions were also prepared and found to produce compounds which were active at GlyT2 with IC_50_ values ranging from 0.81 to 3.5 μM [85]. Interestingly, the C16 lipids presented lower affinity but higher maximal inhibition relative to the C18 lipids (C18 analogues maximal inhibition = 52–67%; C16 analogues maximal inhibition = 88–92%). Further contraction of the lipid chain to C14 also reduced GlyT2 affinity but with a generally increased maximal level of inhibition relative to the C18 lipids. Thus, the order of potency for the lipid analogues tested was C18 > C16 > C14, suggesting that there exists an optimal size and/or configuration of the lipid tail that can be accommodated in the allosteric inhibitor binding pocket. The authors proposed that lipids containing longer C18 tails present additional van der Waals contacts that may engage in more hydrophobic interactions and lead to more potent inhibition [85]. In addition, despite the differences in composition of the lipid tail for the aforementioned analogues and large variation in respective potency values (0.32−9.2 μM), all of these NAGly-derived inhibitors were reversible. These observations are in contrast to OLCarn and suggested that the structural features of head group and not the lipid tail are playing a critical in determining the rate of GlyT2 reversibility. Lastly, OLCarn, 10, 11, and 12 did not deplete synaptic glycine release over time nor did they increase glycine mediated excitatory signaling through NMDA receptors in rat spinal cord slices in vitro [85].

The acyl-glycine SAR findings led to a subsequent SAR campaign whereby the head group of the bioactive lipid scaffold was replaced with a range of aliphatic, aromatic, polar, and ionizable l- and d-amino acids while maintaining the oleoyl tail [86]. All of the analogues containing an l-amino acid head group were found to be active GlyT2 inhibitors, with IC_50_ values ranging from 25.5 nM to 4.35 μM. The basic l-amino acids provided the most potent inhibitors within this series, which included l-His (13; GlyT2 IC_50_ = 130 nM), l-Arg (14; GlyT2 IC_50_ = 47.9 nM), and l-Lys (15; GlyT2 IC_50_ = 25.5 nM; 91% maximal inhibition). These positively charged *N*-acyl amino acids inhibited glycine transport in the range of 71–95%. The exquisite potency exhibited by l-Lys analogue 15 led to the preparation of a focused sample set of its derivatives which included contraction of the lysine alkyl chain, removal of the carboxylic acid, or conversion of the carboxylic acid to the methyl ester and the inhibitory activity decreased relative to parent 15 for all of these modifications. Matched-pair analysis of the d-amino acid congeners revealed that the l-amino acids largely provided more potent inhibitors, with the exception of d-serine. The d-Lys analogue of 15 (16) is approximately 2-fold less potent (16 GlyT2 IC_50_ = 48.3 nM). The authors note that the optimal head groups for analogues within this series contain positively charged or aromatic motifs, which suggests this region of the GlyT2 binding site that the bioactive lipids occupy presents aromatic residues capable of engaging in π-cation and π–π binding interactions [86].

Oleoyl-l-lysine analogue 15 (OLLys) was further assessed for its mechanism of binding at GlyT2 by measuring I_max_ of glycine transport in the presence of increasing concentrations of the inhibitor. The I_max_ of transport was significantly reduced for all concentrations of 15 and the EC_50_ values for glycine were unchanged compared to glycine transport in the absence of any inhibitor, indicating that 15 is a noncompetitive inhibitor of GlyT2. An Eadie−Hofstee plot of the data suggested a mechanism of binding that is not purely noncompetitive, indicating that allosteric lipid binding is impacting glycine binding at the substrate site [86]. An in vitro metabolic stability analysis of 15 with human and rat plasma and liver microsomes showed that the compound is susceptible to rapid metabolism, largely attributed to amide bond hydrolysis (t_1/2_ = 54 min). Conversely, the d-Lys congener 16 (ODLys) was minimally degraded, likely due to the unnatural amide acid configuration reducing hydrolytic cleavage of the amide bond. In light of its superior metabolic stability, oleoyl-d-lysine 16 was furthered screened and was not found to present limiting off-target pharmacology. Pharmacokinetics (PK) studies with 16 in male Sprague Dawley rats receiving a single dose (27.5 mg/kg, i.p.) revealed that the compound was rapidly adsorbed with a T_max_ at 1 h and slowly cleared with a terminal half-life (t_1/2_) of 10 h. Oleoyl-d-lysine 16 was rapidly taken up into the brain and the B/P ratio increased over the sampling period from 0.08 at 1 h to 1.1 at 24 h, and a time averaged B/P ratio of 0.31 was calculated from the AUC_0__–24 h_. The total brain C_max_ was 1854 ng/g (∼4.5 μM) and the calculated unbound drug concentration (*f*_u, brain_) was approximately 90 nM, as per a separate tissue binding assay conducted with rat brain homogenate (99.98% bound). The calculated *f*_u, brain_ of 90 nM for 16 exceeded the GlyT2 IC_50_ (48.3 nM), indicating that systemic administration could produce unbound concentrations within the CNS required for analgesia [59]. Thus, the analgesic efficacy of 16 was next assessed in the PNL model of neuropathic pain. Male Sprague Dawley rats received a single intraperitoneal bolus of 16, and the efficacy in reversing allodynia (i.e., mechanical paw withdrawal threshold (PWT) was taken using the von Frey test) was assessed over 6 h. A 30 mg/kg dose of 16 produced significant analgesia over the first 90 min after injection compared to vehicle (*p* < 0.001 at 15 and 30 min, *p* < 0.01 at 60 and 90 min; Bonferroni’s multiple comparisons test) while a lower dose of 3 mg/kg alleviated allodynia in a manner similar to the positive control ORG25543 (30 mg/kg). Mild side effects were observed in only one animal 15 min post-injection, where localized pain (abdominal contraction) was evident at the i.p. site of drug delivery [86]. Conversely, more severe side effects were observed in four out of the six animals of the 30 mg/kg ORG25543 dose group, whereby animals remained recumbent and exhibited acute abdominal constriction for up to 60 min, with two animals exhibiting writhing behavior [86]. No side effects were seen in the vehicle-treated animals. Thus, oleoyl-d-lysine 16 produced significantly greater analgesia than ORG25543 at the 30 mg/kg dose, but without inducing severe side effects. The adverse effects observed for ORG25543 are attributed to mechanism-based toxicity due to nearly irreversible binding at GlyT2. As 16 displayed considerably less side effects, the relative reversibility of it was compared with ORG25543 using the aforementioned electrophysiology washout assay. For ORG25543, a transport recovery of 35% was observed after 30 min, suggesting that the compound tightly binds to GlyT2 and presents a very slow off-rate. Conversely, transport following inhibition by oleoyl-d-lysine 16 was completely restored after 25 min, indicating that the compound is reversible and has a faster off-rate than ORG25543 [86]. The differences in these binding profiles may explain why 16 was better tolerated and induced less substantial side effects than ORG25543 at the 30 mg/kg dose. A separate study investigating the analgesic efficacy of 16 with CCI mice was also conducted [87]. Oleoyl-d-lysine 16 produced significant and dose-dependent anti-allodynia upon i.p. administration, reaching a peak at 30 mg mg/kg. Mild side effects were seen on the numerical rating score at the highest dose tested (100 mg/kg), however no severe adverse effects or convulsions were observed [60]. In addition, oleoyl-d-lysine 16 did not cause any respiratory depression, a severe and common side effect associated with opioid analgesics [87]. These results for 16 contrasted with ORG25543, which produced limited analgesia, severe side effects, and toxicity at analgesic or higher doses. The numerical rating score and rotarod scores were maximal or near-maximal for ORG25543 as the drug induced severe convulsions at higher doses. These data, together with the rat PNL data, demonstrate that partial and reversible GlyT2 inhibitor oleoyl-d-lysine 16 can provide significant analgesic effects for neuropathic pain with no side effects within the therapeutic range. The authors note that it is unclear whether the improved side effect profile of 16 relative to ORG25543 is attributed to partial occupancy of the transporter or full occupancy with partial inhibition. However, with high protein binding, it is unlikely that free concentrations of the lipids in vivo are likely to saturate the binding site on GlyT2 [86]. Regardless, the data generated for novel bioactive lipid 16 provide a promising lead in the development of second generation GlyT2 inhibitors capable of providing safe and effective analgesia.

Functional analysis of mutant transporters combined with ligand docking and molecular dynamics (MD) simulations of lipid–transporter interactions were conducted to gain an understanding as to how bioactive lipids such as 16 interact with GlyT2 [88,89]. A single conservative point mutation in extracellular loop 4 (EL4), I545L, resulted in GlyT2 transporters with reduced sensitivity to bioactive lipid inhibitors OLCarn and NAGly, suggesting that they bind at a site that induces significant EL4 conformational changes required for transport. Focus on the EL4 region was also supported by the observation that none of the transporters containing mutations in the extracellular facing vestibule allosteric site display any change in bioactive inhibition compared to WT GlyT2. A GlyT2 site-directed mutagenesis campaign was initiated that focused on changing residues in close proximity to EL4 with the following key criteria in place: (i) the residues should be accessible in the outward-facing conformation; (ii) they should not be conserved between GlyT1 and GlyT2 so as to assess their role in differential selectivity of the inhibitors; (iii) alterations should include aromatic residues to confirm the SAR hypothesis that the most potent bioactive lipids contained positively charged or aromatic head groups due to π-cation and π–π stacking binding interactions; (iv) the residues are in regions that have important conformational roles in the GlyT2 transport cycle should; and (v) mutations to GlyT2 residues are made either to resemble GlyT1, or to remove potential interactions with bioactive lipids but not disrupt the overall transport activity, often using substitutions present in the bacterial homologue, leucine transporter (LeuT) [88,89]. Mutations to a cluster of residues on the extracellular halves of transmembrane domain 5 (TM5) and TM8, and the neighboring EL4, produced transporters that were less sensitive to inhibition by OLCarn, with inhibition only reaching 14.9–29.0% for F428A(TM5), V432A(TM5), Y550L(EL4), P561S(TM8), W563L(TM8), and L569F(TM8) mutants. The EC_50_ values of glycine for these mutant transporters are not significantly different to WT suggesting their mechanism of transport is not impaired [88,89].

Using the site directed mutagenesis results, representative bioactive lipids were subsequently docked into an area that encompassed the extracellular and upper-leaflet embedded regions of TM5, TM8, and EL4 of a GlyT2 homology model that was generated from the nortriptyline bound Drosophila dopamine (*d*DAT) structure [88,89]. The location for docking was selected based on the mutagenesis results and the stabilities of the binding locations were assessed using unrestrained MD simulations. Overall, MD simulations of the docked poses suggest that the lipid inhibitors burrow into a novel extracellular allosteric site, with their tail wedged in a hydrophobic cavity between TM5, TM7, and TM8 while the amino acid head groups remain close to the bilayer/water interface and interact with several aromatic amino acids at the extracellular edges of TM5, TM7, TM8, and EL4 [88,89]. The bioactive lipids adopt a kinked structure with the head group interacting with various aromatic residues while the acyl tail is stabilized by aliphatic residues lining the hydrophobic TM5/7/8 cavity. Notable observations include: (i) the differential effects of the W563L mutation may be due to the contribution of the tryptophan indole π electrons for π–π and π-cation interactions with potent bioactive lipids containing aromatic or positively charged amino acid head groups of the most potent lipid inhibitors [88,89]. MD simulations show that W563 is particularly important for stabilizing head group interactions in the binding site; (ii) access of the bioactive lipids to the TM5/7/8 hydrophobic cavity is influenced by I545 in EL4 where I545 appears to sterically restrict the volume of the acyl chain binding pocket; (iii) all C18 acyl-glycine inhibitors have reduced apparent affinities for the F428A transporter. F428 (TM5) and L569 (TM8) lie just outside the base of the TM5/7/8 cavity and form inter-helical contacts and the mutation of these residues may change the shape and volume of the acyl binding pocket [88,89]. Collectively, the GlyT2 site-directed mutagenesis and MD simulation data have identified a novel extracellular allosteric binding site formed by a crevice between TM5/7/8 and EL4. These data help rationalize existing bioactive lipid SAR, which could be used in structure-based drug design efforts toward the development of next generation of allosteric GlyT2 inhibitors exhibiting partial and reversible binding characteristics.

## 8. ORG25543-Related GlyT2 Inhibitors

**Figure 6:**
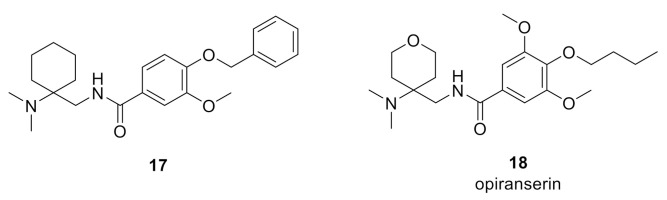
ORG25543-related GlyT2 inhibitors (17) and opiranserin (18).

Mingorance-Le Meur et al. reported slow dissociation of ORG25543 at GlyT2 led to adverse motor effects and a narrow therapeutic index (TI) in the mouse formalin test [90]. The authors confirmed very slow dissociation in a washout timecourse assay similar to the one previously described, which they suggested renders the inhibitor irreversible pharmacologically. In vivo, a maximally tolerated dose (MTD) of 20 mg/kg i.p. induced a phenotype 30 min post-dose consistent with the knockout phenotype, which included convulsions and lethality [90]. A PK-PD mismatch was also observed as the minimally effective dose (MED) (0.06 mg/kg) and MTD were lower than expected as per estimated transporter occupancies based on calculated free drug exposures in brain (estimated occupancies of 6% and 82%, respectively), which is consistent with a slow off-rate and long residence time. Conversely, structurally related analogue 17 (*h*GlyT2 IC_50_ = 100 nM) (Figure 6) presented reversible inhibition with more rapid dissociation in the in vitro electrophysiological washout assay and the compound dose-dependently reduced pain behaviors without adverse effects and with good PK–PD correlation [90]. The MED for 17 (25 mg/kg, i.p.) had an estimated transporter occupancy of 36% and reduced Phase II paw licking behaviors by 33%. A 100 mg/kg i.p. dose produced an 80% reduction in pain behaviors with no convulsions or mortality and the estimated transporter occupancy was 60%. Irwin profile tests were conducted and a total of 53 neurobehavioral and physiological parameters were systematically evaluated before administration and at different timepoints following administration of test compound (5, 15, 30, 60, and 120 min). The parameter changes were grouped into 5 main system activities (central activity, central reactivity, neuromotor tone, neurovegetative reflexes, and autonomic system) [91]. The tests revealed that ORG25543 has a narrow TI and induced tremors and stereotypies at analgesic doses (0.2 and 2 mg/kg) and presented an overall excitatory profile. Contrarily, 17 did not induce tremors or stereotypies at all analgesic doses tested (3, 25, and 100 mg/kg) and presented a sedative profile at the highest dose (100 mg/kg) that was comparable to gabapentin [90]. These findings, in addition to the findings reported for bioactive lipid 16, provide further compelling evidence that a GlyT2 inhibitor with low target residence time or fast dissociation kinetics GlyT2 can provide robust analgesia without adverse effects.

The most advanced GlyT2 inhibitor to date is opiranserin (VVZ-149, 18), a structurally-related analogue of ORG25543 developed by Vivozon, Inc. [92,93]. Opiranserin exhibits modestly potency at GlyT2 (*h*GlyT2 IC_50_ = 0.86 µM) as well as purine P2X_3_ receptor antagonist activity (*h*P2X_3_ IC_50_ = 0.87 µM) and 5-HT_2A_ antagonist activity (*h*5-HT_2A_ IC_50_ = 1.3 µM). GlyT2 reversibility and binding kinetics profile for the compound have not been disclosed. The polypharmacological profile is hypothesized to produce synergistic analgesic effects, which may account for the compounds reported significant preclinical in vivo efficacy despite the moderate potency for each target. Indeed, opiranserin produced an ED_50_ = 20 mg/kg (s.c.) in the rat formalin model (with efficacy observed for a 25 mg/kg s.c. dose comparable to a 3 mg/kg (s.c.) dose of morphine) and an ED_50_ = 80 mg/kg (p.o.) in the rat spinal nerve ligation SNL (Chung) model. Good PK–PD correlations were reported in both studies [92,93].

Opiranserin is the first GlyT2 inhibitor to reach clinical trials and it has been granted fast track status by the FDA. Single ascending dose (SAD) and multiple ascending dose (MAD) Phase 1 clinical trials were conducted with healthy volunteers (NCT02333318, NCT01905410). The drug was administered via a 4-h intravenous infusion of 0.25–8 mg/kg VVZ-149 or placebo in the SAD study (*n* = 46) or a 4-h intravenous infusion of 4–7 mg/kg VVZ-149 or placebo twice daily for 3 days in the MAD study (*n* = 20) [91]. The clinical therapeutic concentration range of VVZ-149 including its active metabolite (VVZ-368) was expected to be 600–1900 μg/L, according to the preclinical study results. The study showed that opiranserin exhibited linear pharmacokinetic characteristics, however, serial blood and urine samples revealed that opiranserin is metabolized to an active metabolite (VVZ-368, structure not disclosed) and that a dose-proportional increase in plasma exposure to opiranserin and VVZ-368 was observed [91]. In addition, a loading dose followed by the continuous i.v. infusion of opiranserin was found to be the most appropriate regimen for maintaining the target therapeutic concentration. Several randomized, double-blind, and placebo-controlled exploratory Phase 2 proof-of-concept trials were conducted to investigate the compound’s ability to treat post-operative pain. These include trials with early gastric cancer patients following laparoscopic-assisted abdominal gastrectomy (NCT02522598), laparoscopic-assisted gastrectomy (NCT02844725), laparoscopic colorectal surgery (NCT02489526, NCT02992041), total hip arthroplasty (NCT03347266), bunionectomy (NCT03997812), lumbar radiculopathy (sciatica) (NCT02644421), and abdominoplasty (NCT03997838). Results have been disclosed for the laparoscopic-assisted abdominal gastrectomy study NCT02844725, which involved 60 patients randomly assigned to receive a 10 h i.v. infusion of opiranserin injections or placebo, initiated approximately 1 h before completion of surgical suturing [94]. Major outcomes for the study included pain intensity and opioid consumption. Furthermore, treatment efficacy of opiranserin was also examined in a subpopulation requiring early rescue medication, which is associated with preoperative negative affect. The study reported that pain intensity was lower in the opiranserin group (*n* = 30) versus the placebo group (*n* = 29), reaching statistical significance at 4 h post-emergence (*p* < 0.05), with a 29.5% reduction in opioid consumption for 24 h and fewer demands for patient-controlled analgesia [94]. In the rescued subgroup, opiranserin further reduced pain intensity (*p* < 0.05) with 32.6% less opioid consumption for 24 h compared to placebo patients. Vivozon is currently recruiting patients for a Phase 3 trial to evaluate the safety and analgesic efficacy of opiranserin injections in post-operative patients following bunionectomy (NCT04430088).

## 9. Miscellaneous GlyT2 Inhibitors

**Figure 7:**
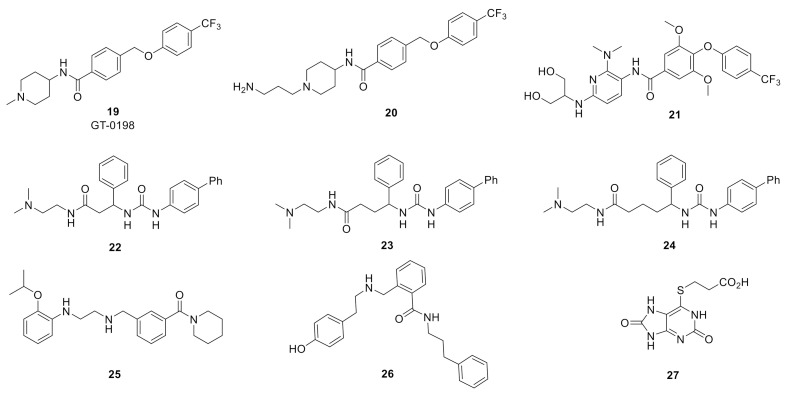
Miscellaneous GlyT2 inhibitors.

GT-0198 (19) [95], developed by Tory Industries, is a selectively GlyT2 inhibitor (*h*GlyT2 IC_50_ = 105 nM; [^3^H]-glycine uptake in stably transfected HEK-293 cells expressing *h*GlyT-2) was derived from early lead phenoxylmethylbenzamide 20 [96] (Figure 7). GT-0198 exhibited dose-dependent analgesic effects in the mouse PSNL neuropathic pain model (p.o. or i.t.). The highest doses administered (30 mg/kg, p.o. and 100 µg, i.t., respectively) produced a comparable analgesic response to a 10 mg/kg (p.o.) and 10 µg (i.t.) dose of gabapentin. Furthermore, the analgesic activity of GT0198 could be antagonized via co-administration of strychnine [95].

Imam and colleagues assessed the analgesic efficacy and tolerability of GlyT2 inhibitor 21, a 3-pyridyl amide derivative of ORG25543, in a rat prostate cancer-induced bone pain (PCIBP) model [97]. The authors reported that 21 is a potent inhibitor (GlyT2 IC_50_ = 2 nM) with good oral exposure (%F = 57%), however, no additional PK and CNS exposure data or in vitro ADME profile information was provided for the compound. PCIBP was induced following intra-tibial injection (ITI) of rat prostate cancer (AT3B) cells into the left tibia of male Wistar Han rats [97]. The animals developed mechanical allodynia in the ipsilateral hind paws and analgesic efficacy upon oral administration of GlyT2 inhibitor 21 (3, 10, and 30 mg/kg), pregabalin (3, 10, 30, and 100 mg/kg), duloxetine (3, 10, 30, and 100 mg/kg), was assessed using von Frey filaments [97]. GlyT2 inhibitor evoked partial pain relief at the 10 and 30 mg/kg doses without any discernible behavioral side effects. By contrast, oral administration of 30 mg/kg of pregabalin induced complete alleviation of mechanical allodynia. None of the doses of duloxetine produced an analgesic response.

Johnson and Johnson reported the discovery of an early series of α-amino acid [98], β and γ-amino acid [99], and benzoylpiperidine [100] GlyT2 inhibitors as exemplified by early inhibitors 22, 23, 24, and 25, respectively. The medicinal chemistry campaigns for these series produced potent GlyT2 inhibitors, however, no in vitro ADME or in vivo analgesic efficacy was reported and further development appears to have been abandoned. Similarly, Pharmacopeia reported SAR studies that led to the discovery of a novel series of potent and selective GlyT2 inhibitors such as 26, however no further development has been disclosed [101].

The recently reported mercaptopurine 27 was discovered via a virtual structure-based pharmacophore screening using free energy perturbation (FEP+) calculations and MD studies using a GlyT2 homology model constructed from the *d*DAT (PDB ID: 4XPT) and human serotonin (SERT)(PDB ID: 5I6X) transporters [102]. The authors proposed that 27 binds at the ligand binding domain (substrate site) and coordinates with sodium and that additional SAR optimization efforts are currently ongoing.

## 10. Augmented Anti-Allodynia Effects upon Co-Administration of GlyT1 and GlyT2 Inhibitors

**Figure 8:**
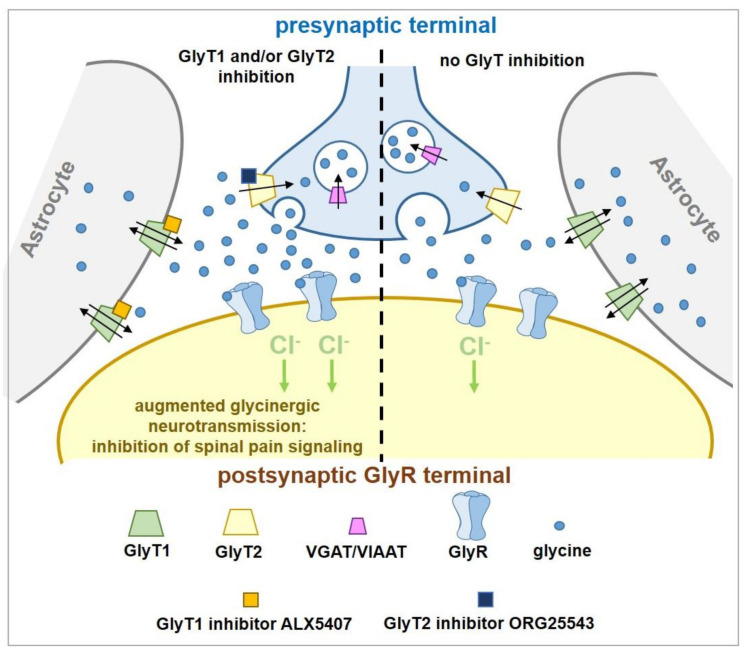
Potential functional consequences of dual GlyT2 inhibition at an inhibitory glycinergic synapse. Shown on the left is a putative synergistic effect of co-application of sub-analgesic doses of ALX5407 (1 and 2 mg/kg, s.c.) and ORG25543 (2 mg/kg, s.c.). In addition to exhibiting a lack of efficacy in the rat PSNL model, the indicated sub-analgesic doses of ALX5407 or ORG25543 did not increase CSF glycine levels. However, co-application of ALX5407 (1 mg/kg, s.c.) and ORG25543 (2 mg/kg, s.c.) lead to significant analgesic activity coupled with robust elevations in CSF glycine. Minimal occupancy and subtle prohibition of glycine uptake by both compounds at their respective GlyTs may produce a synergistic effect, leading to significant increases in synaptic glycine concentrations and augmented inhibitory glycinergic signaling in the spinal dorsal horn. Further investigation into the analgesic efficacy of either combinations of reported GlyT1 and GlyT2 inhibitors devoid of adverse effects or the development of bispecific GlyT1/GlyT2 inhibitors is warranted.

Al-Khrasani and coworkers studied the effects of GlyT1 and GlyT2 inhibitor co-administration (ALX5407 and ORG25543) in the rat PSNL ligation model [103,104]. Administration of 4 mg/kg ALX5407 or ORG25543 (s.c.) produced analgesia 30–60 min post-dose, but lower doses of either compound (1 and 2 mg/kg of ALX5407; 2 mg/kg ORG25543, s.c.) failed to produce anti-allodynia up to 180 min after administration. However, co-treatment with sub-analgesic doses of ALX5407 (1 mg/kg, s.c.) and ORG25543 (2 mg/kg, s.c.) produced analgesia at 60 min and thereafter to 180 min (cessation timepoint of the study) [103,104]. Furthermore, the authors reported a statistically significant elevation in PSNL rat CSF glycine levels following the acute treatment with the combination of sub-analgesic doses of ALX5407 and ORG25543. Interestingly, the sub-therapeutic doses of ALX5407 and ORG25543 did not induce a statistically significant elevation in PSNL rat CSF glycine levels when dosed alone. In addition to providing robust analgesia, the combination of GlyT inhibitors did not affect motor or respiratory function [103,104]. This is a key finding as both inhibitors are tight binders for their respective GlyTs and can induce adverse effects at analgesic doses when dosed separately. These data are quite intriguing and warrant further exploration as they could suggest that combining sub-therapeutic doses of selective GlyT inhibitors or developing non-selective, bispecific GlyT inhibitors might have therapeutic value for treating neuropathic pain (Figure 8).

## 11. Reduced Expression of KCC2 and Altered Chloride Extrusion Capacity: Implications for GlyT Inhibitors for Pain

Reduced expression of KCC2 is another mechanism contributing to pathological pain states via altered glycinergic and GABAergic spinal inhibitory signaling [27,105,106,107]. KCC2 maintains neuronal chloride homeostasis by keeping intracellular chloride concentrations low, thereby allowing for chloride influx upon GABA_A_R and GlyR stimulation. Studies have shown that KCC2 expression levels are reduced upon nerve damage, resulting in limited chloride extrusion capacity and an increase in intracellular chloride concentration [27,105,106,107]. This is turn may alter GABAergic and glycinergic input from hyperpolarizing into depolarizing, thus potentially switching GABA and glycine signaling to promote pain development in neuropathic pain models and potentially making GlyT inhibitors pro-algesic. This may seem paradoxical in light of the extensive and compelling reported preclinical evidence showing that GlyT1 and GlyT2 inhibitors have significant therapeutic potential for treating pain. However, the effects of KCC2 down regulation upon nerve damage could explain the timing of analgesic effects for GlyT inhibitors reported by Morita. In that comprehensive study, Morita reported that GlyT1 and GlyT2 inhibitors induced analgesic activity only after 4 days post-surgery in the mouse PSNL model [59]. When ORG25543 was treated before surgery, or 1 and 2 days after surgery, it did not provide analgesia via reduced withdrawal. Amelioration of the reduced withdrawal threshold occurred with ORG255543 after the 4th day post-surgery. Strychnine and bicuculline, ameliorated allodynia at 20 h and 3 days after surgery, whereas they had no effect after 4 days post-surgery in the PSNL model. These observations are in alignment with altered chloride extrusion capacity due to KCC2 down regulation leading to transient pro-algesic GABAergic and glycinergic signaling. Collectively, the current evidence suggests that GlyT inhibitors may lack an analgesic effect in the early stage of allodynia development after nerve injury, but they are capable of producing potent and long-acting analgesic efficacy against established allodynia. This suggests that glycinergic inhibitory neurotransmission may be functioning normally during the maintenance phase of neuropathic pain, irrespective of altered KCC2 expression. Thus, the timing of GlyT inhibitor application appears to be critical for study designs for both preclinical models of neuropathic pain and for future clinical trials with neuropathic pain patients. Further studies to better determine how GlyT inhibitors can produce analgesic effects during the maintenance phase of neuropathic pain regardless of KCC2 expression are warranted.

## Data Availability

Not applicable.
